# Curcumin Attenuates Chronic Unpredictable Mild Stress-Induced Depressive-Like Behaviors via Restoring Changes in Oxidative Stress and the Activation of Nrf2 Signaling Pathway in Rats

**DOI:** 10.1155/2020/9268083

**Published:** 2020-09-17

**Authors:** Dehua Liao, Chuanfeng Lv, Lizhi Cao, Dunwu Yao, Yi Wu, Minghui Long, Ni Liu, Pei Jiang

**Affiliations:** ^1^Department of Pharmacy, Hunan Cancer Hospital, Changsha, 410013 Hunan, China; ^2^Institute of Clinical Pharmacy & Pharmacology, Jining First People's Hospital, Jining, 272000 Shandong, China

## Abstract

Accumulating evidence has demonstrated that oxidative stress is associated with depression. Our present study aimed at investigating the antidepressant effect and the possible mechanisms of curcumin (CUR) in chronic unpredictable mild stress- (CUMS-) induced depression model in rats. After exposure to CUMS for four weeks, the rats showed depressive-like behavior, and the depressive-like behaviors in CUMS-treated rats were successfully corrected after administration of CUR. In addition, CUR could effectively decrease protein expression of oxidative stress markers (Nox2, 4-HNE, and MDA) and increase the activity of CAT. CUR treatment also reversed CUMS-induced inhibition of Nrf2-ARE signaling pathway, along with increasing the mRNA expression of NQO-1 and HO-1. Furthermore, the supplementation of CUR also increased the ratio of pCREB/CREB and synaptic-related protein (BDNF, PSD-95, and synaptophysin). In addition, CUR could effectively reverse CUMS-induced reduction of spine density and total dendritic length. In conclusion, the study revealed that CUR relieves depressive-like state through the mitigation of oxidative stress and the activation of Nrf2-ARE signaling pathway.

## 1. Introduction

As one of the most common neuropsychiatric illness, depression has affected 300 million people of all ages in the modern world [1]. According to the WHO's prediction, depression is expected to become the world's second leading cause of disability by 2020 [2], leading to a huge social and economic burden on the modern society [3]. In currently clinical practice, many chemical treatments are used for depression, such as tricyclic antidepressants, monoamine oxidase inhibitors, and selective serotonin reuptake inhibitors [4, 5]. However, the existing treatments were not effective to all patients [6] and also accompanied with unwanted side effects [7, 8]. Thus, it is necessary to develop a more effective and safer pharmacological intervention.

Increasing evidence suggested that oxidative stress is responsible for the development of depression [9]. Oxidative stress mainly focused on brain which has a limited amount of antioxidant capacity [10]. It was reported that antidepressants could effectively reduced oxidative damage in depressed patients [11, 12]. The antioxidant subjects like polyphenolic compounds exhibit antidepressant activity in experimentally induced depression models by modulating the brain oxidative stress status [13].

Oxidative stress is an etiologic factor in depressive/neurodegenerative disorders that it is often accompanied by deregulation of nuclear factor erythroid-2-related factor 2 (Nrf2) pathway, a key antioxidant mechanism indicated as a promising target for treatment of depression [14]. As a pivotal transcription factor, Nrf2 was involved in the regulation of the antioxidant response in the brain. Under oxidative stress circumstances, Nrf2 isolate from Kelch-like ECH-associated protein 1 (Keap1) and translocate from cytoplasm into the nucleus [15, 16]. Furthermore, antioxidant response element (ARE) could be upregulated after the activation of the Nrf2 and finally regulates the expressions of a variety of antioxidant enzymes like heme oxygenase-1 (HO-1) and NADPH: quinine oxidoreductase-1 (NQO-1) [17, 18]. Buendia et al. [19] has reported that Nrf2-ARE pathway is proved to reduce oxidative stress and neuroinflammation and play a protective role in neurodegenerative diseases.

Numerous evidence has indicated that depression was associated with a range of changes in synaptic form [20, 21]. Cyclic AMP response element-binding protein (CREB) was a major transcription factor involved in the regulation of genes associated with synaptic and neural plasticity. As an important neurotrophic factor, brain-derived neurotrophic factor (BDNF) supports growth and survival of neurons. Recent study has showed that CREB-BDNF signaling pathway in hippocampus was closely related to depression and the pathogenesis of cognitive function impairments [22]. PSD-95 and synaptophysin were postsynaptic marker and presynaptic marker, respectively, which play an important role in the maintenance of synaptic plasticity.

Curcumin (CUR) is the major active component extracted from *Curcuma longa*, which exhibited anti-inflammatory, antioxidant, immunomodulatory, and neuroprotective activities [23, 24]. It has been increasingly recognized that CUR has the potential to cross blood brain barrier and exert antidepressant-like action. Xu et al. have reported that chronic administration of CUR produced a significant antidepressant property in the treatment of depression in mice model [25]. Notably, CUR's antioxidative properties hold a great deal of potential for the treatment of depression. More and more evidences also showed that the activation of Nrf2 was the main mechanism of CUR in the treatment of oxidant stress-related diseases [26, 27]. Shen et al. have reported that CUR exerts its chemopreventive effects via the induction of antioxidant enzymes by activating Nrf2-ARE signaling [28]. Oxidative stress in the diabetic rat-induced by STZ could be attenuated by CUR through activation of the Keap1-Nrf2-ARE signaling pathway [29]. In addition, CUR augments the cardioprotective effect of metformin in an experimental model of type I diabetes mellitus via the Nrf2/HO-1 pathway which is also reported in previous study [30]. However, the detailed mechanism underlying the antidepressant effects of CUR as related to Nrf2 in the brain remains seldom studied.

Therefore, in our present study, we aimed to investigate the antidepressant-like effect of CUR in CUMS-induced rats. In addition, to further investigate the possible molecular mechanisms underlying the therapeutic effects of CUR, we also assessed whether the possible antidepressant-like effects of CUR are associated with oxidative stress status and the changes on the activation of Nrf2 in the brain.

## 2. Materials and Methods

### 2.1. Animals

Male Sprague-Dawley rats (180-220 g) were provided by the Hunan Cancer Hospital Animal Centre. The rats were housed in standard conditions (23 ± 2°C, 12 h light/dark cycle). Except prior to sucrose preference test (SPT), food and water were freely available in the whole experiment. This study was approved by the Animal Care and Use Committee of Hunan Cancer Hospital (protocol number 016/2018). All experiments were performed in accordance with the Guide for Care and Use of Laboratory Animals (Chinese Council).

### 2.2. CUMS Procedure

The rats were adapted for one week and the CUMS procedure was performed for four weeks as previously described [31]. 24 h food deprivation followed by 24 h water deprivation, 45° cage tilting for 24 h, restraint for 4 h in an empty water bottle, 20 min of noise, 1 min tail clamping, and damp bedding were selected as stressors in our study. All stressors were applied individually, continuously, and randomly, so that the stress procedure is unpredictable. The detailed information for specific modeling methods of CUMS are shown in Table 1.

### 2.3. Experimental Design

Rats were randomly divided into three groups (*n* = 8): control, CUMS, and CUMS+CUR. CUR (suspended in 0.5% Tween 80, purchased from Sigma Chemical Co., USA) was administrated by oral gavage (100 mg/kg/day) in the CUMS+CUR group, and the rats in control group were treated with the same volume of saline.

**Figure 1:**
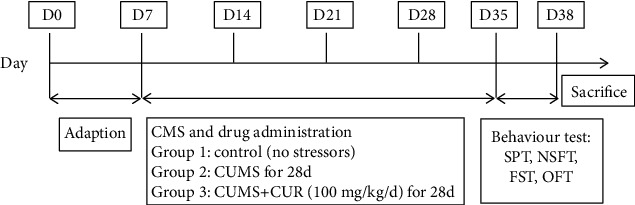
Schematic representation of experimental protocol.

At the end of four weeks, behavioral tests were carried out, and the rats were sacrificed under anesthesia with an intraperitoneal injection of 1% sodium pentobarbital (50 mg/kg). Blood samples and the hippocampus were collected in our study. The whole experimental protocols are shown in Figure 1.

### 2.4. Behavioral Test

#### 2.4.1. SPT

The SPT was performed as our previous study [32]. Prior to testing conditions, all the rats were separated in 1 cage each and habituated to 48 h of forced 1% sucrose solution consumption in two bottles on each side. After deprivation of water for 14 h, two preweighted bottles containing 1% sucrose solution and tap water were given to each rat. After 1 h, the bottles were weighed again, and the consumed weights of 1% sucrose solution and tap water were recorded. The percentage preference for sucrose was calculated as follows: sucrose preference (%) = sucrose consumption/(sucrose consumption + water consumption).

#### 2.4.2. Forced Swimming Test (FST)

The FST was performed as described previously [33] with minor modifications. In brief, rats were separated and were forced to swim in an open cylindrical container (45 cm height, 25 cm diameter) containing 35 cm of water (24 ± 1°C) for a 15 min pretest. The rats were then dried and removed from their cages. 24 hours later, the rats were exposed to the same experimental conditions outlined above for a 5 min FST. Immobility time was scored by an experienced observer blind to the experiment design, defined as floating with only small movement necessary to keep the head above water.

#### 2.4.3. Novelty-Suppressed Feeding Test (NSFT)

All the rats were food deprived for 24 h in their home cages before NSFT. A piece of white paper (10 × 10 cm) was placed in an open field (75 × 75 × 40 cm), and a small amount of food was placed on this paper. The rats were allowed to explore the open field for 8 min. The time it took for the rat to approach and take the first bite of the food was defined as the latency time and was recorded in our study. Immediately afterwards, the animals were transferred to their home cage, and the total food intake for the next 5 min was also weighed to avoid the influence of the animals' appetite.

#### 2.4.4. Open Field Test (OFT)

The open field apparatus consisted of a 76 × 76 cm gray wooden box with 42 cm high boundary walls. The floor was divided into 25 equal squares by black lines. Each rat was placed in the center of the square and left to explore it freely for 5 min. The number of crossing and rearing was recorded by the observer blind to the treatment condition of the animal. The apparatus was cleaned with ethanol and water to remove olfactory cues.

### 2.5. Determination of Serum Corticosterone

For the determination of serum corticosterone, the blood samples were collected at 13:00-15:00 on day 38 before sacrifice. The collected plasma was centrifuged (3500×g, 15 min) at 4°C and stored at -80°C until analysis. The serum corticosterone levels were measured using a commercial ELISA kit (Cayman Chemical, USA) according to the manufacturer's instructions. The standards and samples were all run in duplicates, and the averaged data were used for statistical analysis.

### 2.6. Real-Time PCR Analysis

According to the instruction of manufactory, total RNA was extracted from the hippocampus using TRIzol reagent (Invitrogen Corp., Carlsbad, CA, USA). The mRNA expression of Nrf2, NQO-1, and HO-1 was determined in our present study. Quantitative PCR was performed on Bio-Rad Cx96 Detection System (Bio-Rad, Hercules, CA, USA) using the SYBR green PCR kit (Applied Biosystems Inc., Woburn, MA, USA) and gene-specific primers. The sequences of gene-specific primers are listed in Table 2. A 5 ng cDNA sample was used with 40 cycles of amplification. Each cDNA was determined in triplicate. The signals were normalized to *β*-actin as an internal standard.

### 2.7. Determination of Antioxidant Enzyme Activities and Lipid Peroxidation

Malondialdehyde (MDA) content was determined according to the previous report [34]. Briefly, 1 ml of 15% trichloroacetic acid was added to 500 *μ*l of brain homogenate supernatant and mixed well, and then, the solutions were centrifuged at 1006×g for 10 min. One milliliter of the supernatant was added to 0.5 ml of 0.7% TBA, and then, the mixture was heated for 60 min at 90°C. The absorbance was recorded at 532 nm using UV spectrophotometer. CAT activity was assayed by H_2_O_2_ consumption, following Aebi's [35].

### 2.8. Western Blot Analysis

For western blot analysis, total protein was prepared from the hippocampus, and the Bradford method was used to determine its concentration. The hippocampus sample was loaded on a precast 12% SDS-PAGE gel with 10 *μ*g proteins in each lane. Proteins in the gels were transferred to a polyvinylidene fluoride membrane and blocked for 1 h in 5% nonfat dry milk in TBS-T (25 mM Tris, pH 7.5, 150 mM NaCl, 0.05% Tween-20). The antibodies and concentrations listed below were used overnight at a temperature of 4°C: Nox2 (Santa Cruz; sc-130549; 1 : 800); 4-HNE (Abcam ab48506; 1 : 1000); Nrf2 (Abcam; ab137550; 1 : 200); pCREB (Cell Signaling; 9198; 1 : 1000); CREB (Cell Signaling; 9197; 1 : 1000); BDNF (Abcam; ab108319; 1 : 2000); PSD-95 (ProteinTech; 20665-1-AP; 1 : 2000); synaptophysin (ProteinTech; 17785-1-AP; 1 : 1000); PCNA (ProteinTech; 10205-2-AP; 1 : 3000); and *β*-actin (ProteinTech; 60008-1-Ig; 1 : 4000). Membranes were then probed with horseradish peroxidase-conjugated secondary antibody for 40 min. After washing, the membranes were dipped in electrochemiluminescence, and immunoblots were analyzed by using the Bioprofl Biolight PC software (Vulber Lourmat, France). *β*-Actin was used as an internal standard to normalize the signals.

### 2.9. TUNEL Staining

According to the manufacturer's instructions, the terminal deoxynucleotidyl transferase-mediated deoxyuridine triphosphate nick-end labeling (TUNEL) detection kit (KeyGen Biotech, Nanjing, China) was used to assess apoptosis. Apoptotic index was defined as the average percentage of TUNEL-positive cells in 20 nonoverlapping cortical fields under ×200 magnification.

### 2.10. Immunohistochemical Staining

Paraffin-embedded tissue sections were rehydrated first in xylene and then in graded ethanol solutions. The slides were then blocked with 5% bovine serum albumin (BSA) in Tris-buffered saline (TBS) for 2 h. After incubation with anti-8-OHDG and anti-Nox2 overnight at 4°C, the sections were then washed with PBS and incubated with secondary antibodies. Counter staining was performed using hematoxylin, and the slides were visualized under a light microscope.

### 2.11. Golgi Staining

Golgi staining was performed as previous report [36]. In brief, the brain tissues of the rat were kept in the Golgi-Cox solution for 14 days in the darkness, and the solution was replaced every 48 h. After dehydration with 30% sucrose solution, the tissues were cut into 100 *μ*m section. The following steps included treatment with ammonia water and acid hardening fixing bath and dehydration with increasing concentrations of alcohol. A digital camera attached with microscopy was used to take images of the tissues for dendritic structure analyzing. Dendritic spine density and total dendritic length analysis were done manually using the Fiji software under ×400 magnification.

### 2.12. DHE Staining

Reactive oxygen species (ROS) was measured by dihydroethidium (DHE) microfluorography as previous study [37]. In brief, freshly prepared frozen brain sections (15 *μ*m thick) were incubated with 5 *μ*M DHE in PBS at 37°C for 30 min in a dark humidified chamber. The sections were then imaged by using the Leica fluorescence microscope (Leica Microsystems, Germany).

### 2.13. Statistical Analysis

Statistical Package for Social Science (SPSS) version 18 (SPSS Inc., Chicago, IL, USA) was used for data analysis in our study. All data were analyzed by one-way analysis of variance (ANOVA) with least significant difference (LSD) post hoc multiple comparisons. All data were presented as means ± SD, and *p* < 0.05 was considered statistically significant.

## 3. Results

### 3.1. Effects of CUR on Behavioral Tests

**Figure 2:**
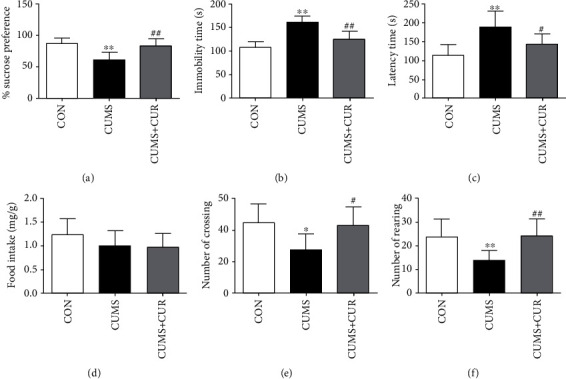
Effect of CUR on CUMS-induced behavior changes. (a) Sucrose preference in SPT, (b) immobility time in FST, (c) latency time in NSFT, (d) food intake in NSFT, (e) number of crossing in OPT, and (f) number of rearing in OPT. Data are expressed as means ± SD (*n* = 8). ∗*p* < 0.05 and ∗∗*p* < 0.01 compared to the control group. ^#^*p* < 0.05 and ^##^*p* < 0.01 compared to the CUMS group.

The CUMS group showed reduced source preference in SPT (Figure 2(a), *p* < 0.01), prolonged immobility time in FST (Figure 2(b), *p* < 0.01), and latency time in NSFT (Figure 2(c), *p* < 0.01) in comparison with the rats in control group. Our study also observed that the number of crossing (Figure 2(e), *p* < 0.05) and rearing (Figure 2(f), *p* < 0.01) in OPT was all significantly decreased in the CUMS group. In comparison with the CUMS group, the administration of CUR successfully increased the sucrose preference (Figure 2(a), *p* < 0.01), decreased immobility time (Figure 2(b), *p* < 0.01) and latency time (Figure 2(c), *p* < 0.01), and increased the number of crossing (Figure 2(e), *p* < 0.05) and rearing (Figure 2(f), *p* < 0.01) in the CUMS+CUR group. In addition, no significant difference of food intake was observed in NSFT.

### 3.2. Effects of CUR on Corticosterone Level

**Figure 3:**
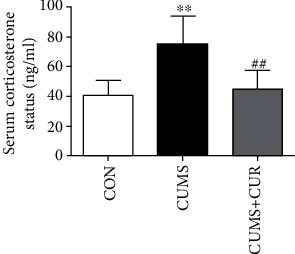
Effect of CUR on CUMS-induced serum corticosterone level. Data are expressed as means ± SD (*n* = 8). ∗*p* < 0.05 and ∗∗*p* < 0.01 compared to the control group. ^#^*p* < 0.05 and ^##^*p* < 0.01 compared to the CUMS group.

As displayed in Figure 3, the serum corticosterone level significantly increased (*p* < 0.01) in the CUMS group compared with the control group. However, the administration of CUR markedly decreased (*p* < 0.01) the corticosterone level when compared with the rats in the CUMS group.

### 3.3. Effect of CUMS and CUR on Oxidative Stress

**Figure 4:**
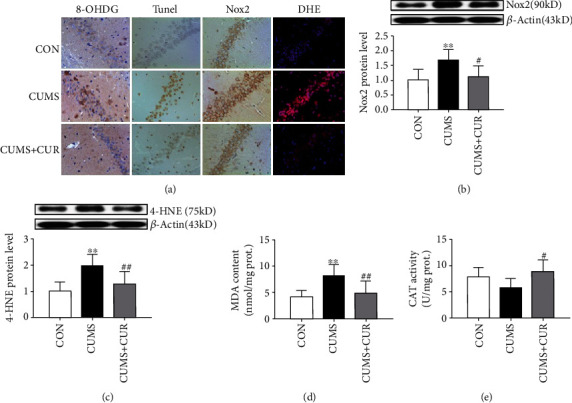
Effects of CUR on CUMS-induced oxidative stress change. (a) Immunohistochemical staining, TUNEL staining, and DHE staining, (b) protein expression of Nox2, (c) protein expression of 4-HNE, (d) content of MDA, and (e) activity of CAT. Data are expressed as means ± SD (*n* = 8). ∗*p* < 0.05 and ∗∗*p* < 0.01 compared to the control group. ^#^*p* < 0.05 and ^##^*p* < 0.01 compared to the CUMS group.

The immunohistochemical staining results of 8-OHDG and Nox2 are shown in Figure 4(a); the results showed that the expressions of 8-OHDG and Nox2 were all increased in CUMS-treated rats when compared to control group, and the supplementation of CUR markedly moderated CUMS-induced increasing of 8-OHDG and Nox2. The results of DHE immunostaining showed that ROS production was significantly increased in the CUMS group, and this increase in ROS generation was markedly alleviated by the pretreatment with CUR (Figure 4(a)). The protein expressions of Nox2 (Figure 4(b), *p* < 0.01) and 4-HNE (Figure 4(c), *p* < 0.01) were significantly increased in the CUMS group as compared to the rats in the control group, and the administration of CUR effectively mitigated CUMS-induced increasing of Nox2 (Figure 4(b), *p* < 0.05) and 4-HNE (Figure 4(c, *p* < 0.01); the western blot result of Nox2 was in accordance with immunohistochemical staining results. The content of MDA (Figure 4(d), *p* < 0.01) was significantly increased in the CUMS group when compared to the control group. The CUR treatment successfully decreased the content of MDA in the CUMS+CUR group when compared to the CUMS group (Figure 4(d), *p* < 0.01). In comparison with the rats in the control group, the activity of CAT (Figure 4(e), *p* > 0.05) was decreased in CUMS-treated rats but without significance difference. Furthermore, the administration of CUR significantly increased CAT activity in the CUMS+CUR group when compared to the CUMS group (Figure 4(e), *p* < 0.05).

### 3.4. Effects of CUR on the Activation of Nrf2 in CUMS-Treated Rats

**Figure 5:**
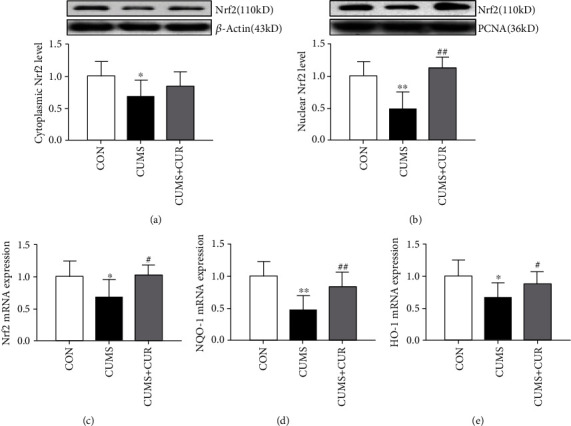
Effects of CUR on the activation of Nrf2 in CUMS-treated rats. (a) Protein expression of Nrf2 in cytoplasmic, (b) protein expression of Nrf2 in nuclear, (c) mRNA expression of Nrf2 in the hippocampus, (d) mRNA expression of NOQ-1in the hippocampus, and (e) mRNA expression of HO-1in the hippocampus. Data are expressed as means ± SD (*n* = 8). ∗*p* < 0.05 and ∗∗*p* < 0.01 compared to the control group. ^#^*p* < 0.05 and ^##^*p* < 0.01 compared to the CUMS group.

The Nrf2 levels in cytoplasmic (Figure 5(a), *p* < 0.05) and nuclear (Figure 5(b), *p* < 0.01) all significantly decreased in the CUMS group when compared with the rats in the normal-treated group, and the Nrf2 in nuclear obviously (Figure 5(b), *p* < 0.01) increased in the CUMS+CUR group compared to the CUMS group. Interestingly, as shown in Figure 5(c), the gene expression of Nrf2 was similar with the protein expression of Nrf2 in the nuclear. The mRNA expressions of NQO-1 (Figure 5(d), *p* < 0.01) and HO-1 (Figure 5(e), *p* < 0.05) all significantly decreased in the CUMS model rats, and CUR treatment significantly prevented the decrease of NQO-1 (Figure 5(d), *p* < 0.01) and HO-1 (Figure 5(e), *p* < 0.05) in the CUMS+CUR group when compared with the rats in the CUMS group.

### 3.5. Effect of CUMS and CUR on Synaptic Plasticity

**Figure 6:**
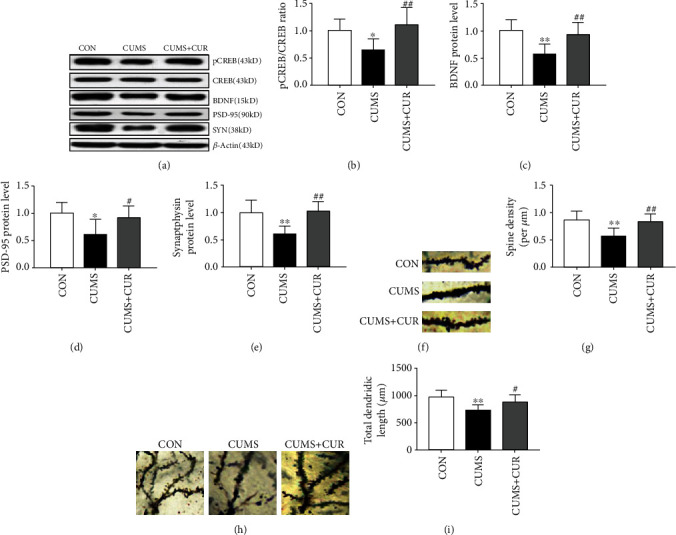
Effect of CUR on synaptic plasticity. (b) pCREB/CREB ratio in the hippocampus, (c) protein expression of BDNF in the hippocampus, (d) protein expression of PSD-95 in the hippocampus, (e) protein expression of synaptophysin in the hippocampus, (f) representative images of dendritic spines from DG granule neurons in the rat, (g) spine density in different groups, (h) representative images of DG granule neurons in the rats, and (i) total dendritic length in different groups. Data are expressed as means ± SD (*n* = 8). ∗*p* < 0.05 and ∗∗*p* < 0.01 compared to the control group. ^#^*p* < 0.05 and ^##^*p* < 0.01 compared to the CUMS group.

As shown in Figure 6(b), the pCREB/CREB ratio was significantly decreased in the CUMS group compared to vehicle control group (Figure 6(b), *p* < 0.05). Administration of CUR significantly increased pCREB/CREB ratio in the hippocampus compared to the CUS-treated rats (Figure 6(b), *p* < 0.01). The protein expression of BDNF (Figure 6(c), *p* < 0.01), PSD-95 (Figure 6(d), *p* < 0.05), and synaptophysin (Figure 6(e), *p* < 0.01) all significantly decreased in the CUMS-treated rats, and CUR successfully reversed the CUMS-induced decrease of these three proteins (*p* < 0.01). Previous studies have reported that dynamic alterations in synaptic and dendritic structure and function play a pivotal role in the development of depression [38, 39]. In our present study, Golgi staining showed that spine density (Figure 6(g), *p* < 0.01) and total dendritic length (Figure 6(i), *p* < 0.01) significantly decreased in the dentate gyrus (DG) granule neurons of CUMS-induced rats, and the administration of CUR markedly reversed this effect (Figure 6(g), *p* < 0.01, Figure 6(i), *p* < 0.05).

## 4. Discussion

Our present study demonstrated that the administration of CUR exhibited antidepressant-like activities in CUMS-induced depression model. We investigated the depressive-like behaviors (SFT, FST, NSFT, and OFT) in rats under CUMS, and chronic administration of CUR normalized behavioral changes in rats exposed to stress. CUR could effectively decrease protein expression of oxidative stress marker (MDA, Nox2, and 4-HNE). CUR could also activate stress-induced Nrf2-ARE axis inhibition. In addition, long-term treatment with CUR markedly prevented CUMS-induced reduction of BDNF, PSD-95, and synaptophysin expressions. These findings indicate the potential benefits of administration of CUR to reverse the development of depression. Furthermore, the antidepressant mechanism of CUR may be mediated by restoring changes in oxidative stress and the activation of the Nrf2-ARE signaling pathway.

The CUMS model has long been used as animal model of depression, and previous study showed that most effects of CUMS could be effectively reversed by antidepressant agents [40]. In our present study, reduced sucrose preference in SPT and prolonged immobility time in FST were observed in the CUMS group, which indicated the depressive-like state. In comparison with the rats in the CUMS group, chronic administration of CUR successfully increased sucrose preference and decreased immobility time, which was consistent with the former studies [41, 42]. Anxiety status was assessed by NSFT and OFT. Our study observed that CUR treatment could successfully reverse CUMS-induced increase in latency time in NSFT and decrease in crossing number and rearing number in OFT. Motaghinejad et al. have also reported that chronic administration of CUR could effectively improve ambulation number and ambulation distance in nicotine-treated rats, indicating neuroprotective effect of CUR against nicotine-induced neurotoxicity [43]. Therefore, CUR exhibited antidepressant-like properties basing on the above-mentioned results.

As a stress marker, corticosterone level is widely used for accessing the stress state. More and more evidences showed that the elevated corticosterone level was associated with depressive-like behaviors, and antidepressant-like activity could always induce a reduction of corticosterone level [44–46]. In the present study, the corticosterone serum level was significantly increased in rats exposed to CUMS model compared to the nonstressed group, and chronic administration of CUR successfully reversed CUMS-induced the elevation of the corticosterone serum level. The results that serum corticosterone in the rats of the CUMS group increased significantly were in accordance with previous studies [2, 47]. As a typical antidepressant agent, fluoxetine could effectively prevent the elevation of serum corticosterone level in CUMS-treated rats [1, 48]. Disrupting the HPA axis and elevating the levels of serum corticosterone by CUMS were increasingly recognized [49, 50]. In addition, CUMS-induced increase of serum corticosterone level may be due to an impaired negative feedback in the HPA [51, 52] and lack of the inhibitory role of the hippocampus in glucocorticoid synthesis [53].

The elevated free radical generation and decreased activity of antioxidants break the balance between oxidant-antioxidant systems, which will always induce oxidative stress [54]. More and more evidence showed that the brain is highly susceptible to oxidative damage [55], and oxidative stress plays a pivotal role in CUMS-induced depression [54]. The release of excitatory amino acid and the expression of specific gene may be enhanced by ROS, which will always induce lipid peroxidation and DNA oxidation, subsequently resulting in neuronal apoptosis [56]. Our present study observed that the intensity of DHE staining and TUNEL-positive cells significantly increased in CUMS-treated rats, which indicated oxidative stress induced a severe neuronal apoptosis. Fortunately, as a natural antioxidant agent, the administration of CUR significantly alleviated CUMS-induced oxidative stress and neuronal apoptosis in the CUMS+CUR group. In our present study, signs of oxidative stress were observed as exemplified by the decrease of antioxidant enzyme activity, such as CAT. In addition, as lipid peroxidation markers, MDA and 4-HNE levels all significantly increased in the CUMS group when compared to the control group. Previous study has reported that CUR has shown to counteract oxidative stress by reducing lipid peroxidation and improving the activity of antioxidant enzymes [54]. Our study results were consistent with this report, which expressed a significantly decrease in MDA and 4-HNE levels and a markedly increase in CAT activity in the CUMS+CUR group. Furthermore, DNA is an important and recognized target of free radicals attack. 8-OHDG is one of the most widely studied biomarkers of oxidative DNA damage. The immunohistochemical staining results coincide with a previous study, which indicated that CUR effectively reversed the increase of 8-OHDG expression under CUMS. ROS and oxidative stress are mainly generated from Nox which is a multiunit enzyme [57]. In particular, the primary mechanism underlying the development of oxidative stress in various neurodegenerative conditions is the activation of Nox2 [58]. In our present study, the protein expression of Nox2 significantly increased in CUMS-treated rats, and the administration of CUR successfully alleviated this phenomenon in the CUMS+CUR group. These results supported that oxidative stress plays a pivotal role in CUMS-induced depression. In addition, our results also demonstrated that the neuroprotective effect of CUR was mediated via its antioxidative ability.

As a transcription factor, Nrf2 is known to play a pivotal role in modulating oxidative stress and exhibiting an important protective role in brain injury and neurodegenerative diseases. As it is known to us, Nrf2 is mainly located in the cytoplasm under physical conditions. However, Nrf2 translocates into the nucleus in response to oxidative stress [15]. In our present study, the results of western blot showed that CUR administration promoted Nrf2 nuclear translocation. This indicates that the Nrf2 level markedly increased in CUMS+CUR-treated rats when compared with the rats in the CUMS group. And the protein expression of Nrf2 was without significant difference in both the CUMS+CUR group and the CUMS group. The above observations revealed that Nrf2 was inhibited when rats exposed to CUMS and CUR had the ability to activate Nrf2. Previous studies have also reported that CUR had the ability to activate Nrf2 and provide neuroprotection from a traumatic brain injury [16, 56]. In order to investigate the regulation effects of CUR on the Nrf2 downstream pathway, the mRNA levels of Nrf2, NQO-1, and HO-1 were evaluated in our study. NQO-1 and HO-1 are important antioxidant enzymes in the Nrf2-ARE pathway [59]. The results showed that chronic administration of CUR could significantly reverse CUMS-induced decrease in the mRNA level of Nrf2, NQO-1, and HO-1. The above results indicated that the Nrf2 downstream pathway was inhibited under CUMS, and chronic administration of CUR could effectively activate this signal pathway. Our results indicated that the Nrf2 signal pathway was inhibited under CUMS, and chronic administration of CUR enhanced Nrf2 translocation from cytoplasm to nucleus and increased expression of antioxidant enzymes through Nrf2 signal pathway, thereby protecting the brain against CUMS-induced depression.

CREB is involved in the regulation of genes associated with synaptic and neural plasticity [43]. CREB has been reported to be phosphorylated by many signaling events on serine 133 [60]. The results in our study showed that the ratio of pCREB/CREB significantly decreased in the CUMS group, and chronic administration of CUR successfully reversed this reduction. These results were in conformity with previous studies which have shown that CUR can increase the pCREB/CREB ratio in rats exposed to CUMS [61]. In addition, a previous study also showed that CUR could function as a potential agent that suppresses depressive-like behavior via the prevention of protein changes associated with synaptic plasticity [62]. As the most abundant neurotrophin in the brain, BDNF plays a crucial role in the regulation of survival as well as synaptic plasticity. Our study investigated whether BDNF was involved in the antidepressant effects induced by CUR. The results indicated that the expression of BDNF in rats exposed to CUMS was significantly increased after chronic administration of CUR. Furthermore, other synapse-associated proteins were also accessed in our study. PSD-95 and synaptophysin were postsynaptic marker and presynaptic marker respectively. In accordance with BDNF, CUR successfully alleviated CMUS-induced reduction in the protein level of PSD-95 and synaptophysin. Zhang et al. have reported that CUR can reverse the decreased expression of BDNF, PSD-95, and synaptophysin in CMS-induced rats [62]. Previous studies have shown that spine densities and synaptic plasticity were closely correlated with the function of neuron and cognitive performance [63, 64]. Our present study shows that CUR could effectively reverse CUMS-induced decrease of spine density and total dendritic length. Hence, the alteration of the above-mentioned synaptic plasticity-associated proteins may underlie changes in functional plasticity associated with CUMS-induced depression.

## 5. Conclusion

In conclusion, our present study suggests that the administration CUR is effective in preventing CUMS-induced depression. Furthermore, the current results suggested that the antidepressant action of CUR may be mediated by restoring changes in oxidative stress, the Nrf2-ARE signaling pathway, and the synaptic and neural plasticity, which might ultimately contribute to its antidepressive-like effect.

## Figures and Tables

**Table 1:** Specific modeling methods of CUMS.

Number	CUMS procedure
1	24 h food deprivation
2	24 h water deprivation
3	45° cage tilting for 24 h
4	Restraint for 4 h in an empty water bottle
5	20 min of noise
6	1 min tail clamping
7	Damp bedding

**Table 2:** Primers used in real-time PCR analyses of mRNA expression.

Gene	Sense primer (5′-3′)	Antisense primer (5′-3′)
HO-1	TGCTCGCATGAACACTCTGGAGAT	ATGGCATAAATTCCCACTGCCACG
NQO-1	GTGAGAAGAGCCCTGATTGT	CCTGTGATGTCGTTTCTGGA
Nrf2	CCCAGCACATCCAGACAG	TATCCAGGGCAAGCGACT
*β*-Actin	CATCCTGCGTCTGGACCTGG	TAATGTCACGCACGATTTCC

## Data Availability

The data used to support the findings of this study are available from the corresponding author upon request.
